# New insights into osmobiosis and chemobiosis in tardigrades

**DOI:** 10.3389/fphys.2023.1274522

**Published:** 2023-10-19

**Authors:** Lykke K. B. Hvidepil, Nadja Møbjerg

**Affiliations:** Department of Biology, University of Copenhagen, Copenhagen, Denmark

**Keywords:** chemobiosis, cryptobiosis, *Echiniscoides sigismundi*, osmobiosis, survival strategies, toxicants, tardigrades, tun

## Abstract

Tardigrades are renowned for their ability to enter the extremotolerant state of latent life known as cryptobiosis. While it is widely accepted that cryptobiosis can be induced by freezing (cryobiosis) and by desiccation (anhydrobiosis), the latter involving formation of a so-called tun, the exact mechanisms underlying the state—as well as the significance of other cryptobiosis inducing factors—remain ambiguous. Here, we focus on osmotic and chemical stress tolerance in the marine tidal tardigrade *Echiniscoides sigismundi*. We show that *E. sigismundi* enters the tun state following exposure to saturated seawater and upon exposure to locality seawater containing the mitochondrial uncoupler DNP. The latter experiments provide evidence of osmobiosis and chemobiosis, i.e., cryptobiosis induced by high levels of osmolytes and toxicants, respectively. A small decrease in survival was observed following simultaneous exposure to DNP and saturated seawater indicating that the tardigrades may not be entirely ametabolic while in the osmobiotic tun. The tardigrades easily handle exposure to ultrapure water, but hypo-osmotic shock impairs tun formation and when exposed to ultrapure water the tardigrades do not tolerate DNP, indicating that tolerance towards dilute solutions involves energy-consuming processes. We discuss our data in relation to earlier and more contemporary studies on cryptobiosis and we argue that osmobiosis should be defined as a state of cryptobiosis induced by high external osmotic pressure. Our investigation supports the hypothesis that the mechanisms underlying osmobiosis and anhydrobiosis are overlapping and that osmobiosis likely represents the evolutionary forerunner of cryptobiosis forms that involve body water deprivation.

## 1 Introduction

Tardigrades are microscopic aquatic animals renowned for their resilience towards harsh environmental conditions, a resilience that relies on their ability to enter dormancy in the form of various resting states, most notably the state of cryptobiosis (Keilin, 1959; Crowe, 1975; Wright et al., 1992; Clegg, 2001; Guidetti et al., 2011; Møbjerg et al., 2011; Erdmann and Kaczmarek, 2017; Guidetti and Møbjerg, 2018; Hibshman et al., 2021; Møbjerg and Neves, 2021). While in the highly resistant state of cryptobiosis, tardigrades express an exceptional tolerance towards extreme fluctuations in abiotic factors and they, among others, endure prolonged periods of desiccation and freezing as well as extremes such as vacuum, very high pressure and extreme levels of radiation (Rebecchi et al., 2007; Rebecchi et al., 2009; Horikawa et al., 2008; Jönsson et al., 2008; Hengherr et al., 2009a; Hengherr et al., 2009b; Nilsson et al., 2010; Persson et al., 2011; Hashimoto et al., 2016; Ono et al., 2016; Jönsson, 2019; Møbjerg et al., 2022; Lee et al., 2022).

Cryptobiosis was defined by Keilin (1959) as the “*state of an organism when it shows no visible signs of life and when its metabolic activity becomes hardly measurable, or comes reversibly to a standstill.*” Keilin (1959) recognized four forms of cryptobiosis induced by different environmental conditions, *i.e.*, anhydrobiosis (dehydration), cryobiosis (cooling), anoxybiosis (lack of oxygen) and osmobiosis (high salt concentrations). A great body of studies have dealt with anhydrobiosis and desiccation tolerance in tardigrades (*e.g.*, Westh and Ramløv, 1991; Jönsson and Rebecchi, 2002; Hengherr et al., 2008; Halberg et al., 2013; Ito et al., 2016; Boothby et al., 2017; Wełnicz et al., 2011; Schill and Hengherr, 2018; Kaczmarek et al., 2019). We hypothesize that anhydrobiotic animals have the ability to sense a decrease in water potential and that this initiates the subsequent series of morphological and biochemical changes, which in tardigrades and bdelloid rotifers involve a contraction of the body into a so-called “tun” (Crowe and Madin, 1974; Ricci et al., 2003; Halberg et al., 2013). Our previous investigations further suggest that muscle protein filaments play a crucial role in sustaining structural integrity, stabilizing the anhydrobiotic tun state (Halberg et al., 2013; Møbjerg et al., 2018; Neves et al., 2020; Møbjerg and Neves, 2021).

Organisms entering anhydrobiosis obviously need to deal with excessive water loss. Loss of intra- and extra-cellular water is, however, also a characteristic of cryptobiosis induced by hyper-osmotic solutions and freezing. We therefore hypothesize that the mechanisms underlying anhydrobiosis initially evolved in the marine environment as a mechanism to withstand variation in temperature and external osmotic concentration (Hygum et al., 2016; Sørensen-Hygum et al., 2018; Møbjerg et al., 2022). Osmobiosis could thus be the evolutionary forerunner of anhydrobiosis and cryobiosis (Emdee et al., 2023). Little is, however, known of osmobiosis, and some authors have argued that this form of cryptobiosis may not be a “true” form of the state (Wright et al., 1992), implying that osmotic stress tolerance involves energy dependent osmoregulation.

Here, we provide a detailed experimental investigation into the phenomenon osmobiosis using the marine intertidal tardigrade *Echiniscoides sigismundi* as a model. This species is highly tolerant of fluctuations in external salt concentration and furthermore exhibits an array of tolerances against other extremes, including complete desiccation, freezing, resilience towards environmental toxicants and high levels of radiation (Clausen et al., 2014; Hygum et al., 2016; Hygum et al., 2017; Jönsson et al., 2016; Sørensen-Hygum et al., 2018; Kamilari et al., 2019).

We show that *E. sigismundi* enters the tun state and readily handles exposure to saturated seawater, providing clear evidence of an osmobiotic response that mimics tun formation during anhydrobiosis. A small decrease in survival is observed following simultaneous incubation in saturated seawater and the mitochondrial uncoupler DNP indicating that the tardigrades may not be entirely ametabolic while in the osmobiotic tun. Interestingly, DNP, on its own, induces transformation into the tun state, when applied to active state tardigrades in locality seawater. The tardigrades regain full activity upon return to seawater without the chemical. The latter provides evidence of chemobiosis, *i.e.*, cryptobiosis induced by environmental toxicants (Møbjerg et al., 2011; Hygum et al., 2017; Møbjerg and Neves, 2021; McCarthy et al., 2022; Brown et al., 2023). We discuss our data in relation to earlier and more contemporary studies on cryptobiosis in tardigrades and stress that osmobiosis should be defined as a state of cryptobiosis induced by high external osmolyte concentrations. Our investigation supports the hypothesis that the mechanisms underlying osmobiosis and anhydrobiosis are overlapping and that osmobiosis could indeed be the evolutionary forerunner of cryptobiosis forms that involve body water deprivation.

## 2 Materials and methods

### 2.1 Tardigrade collection

**FIGURE 1:**
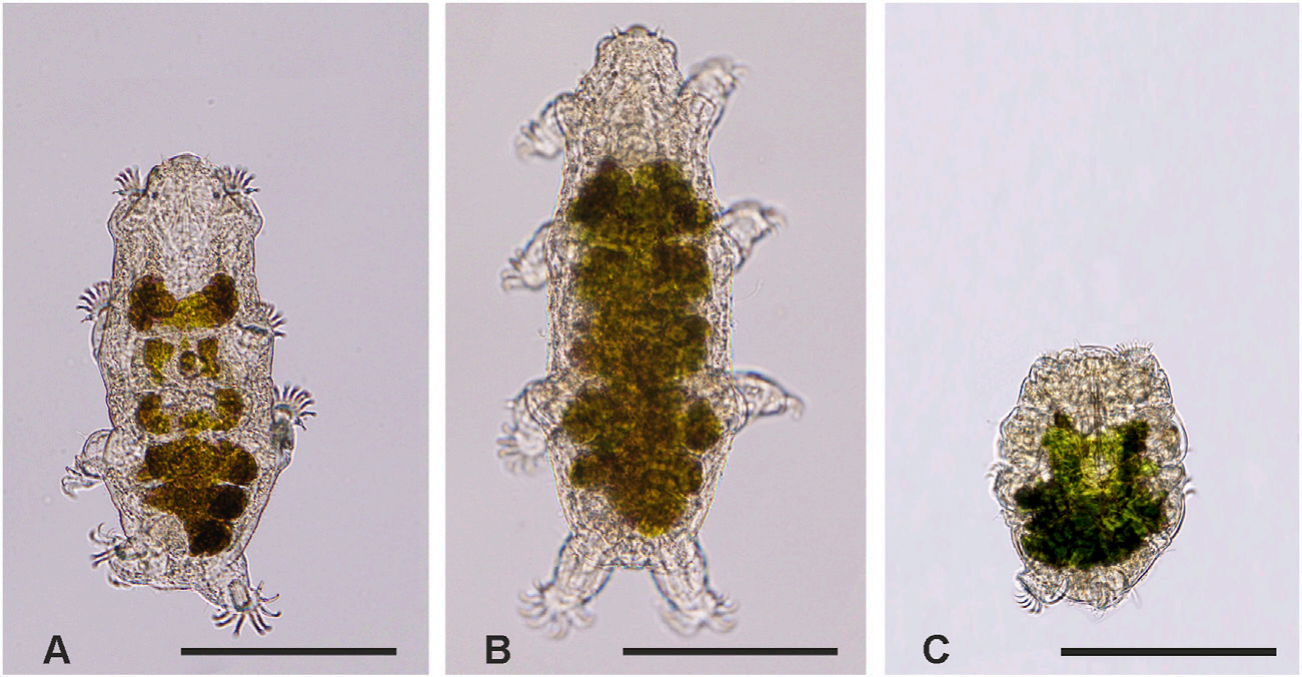
Light micrographs of *Echiniscoides sigismundi.* **(A)** In active state, **(B)** during hypo-osmotic stress (∼0‰), and **(C)** in the hyper-osmotic induced (∼240‰) tun state. Scale bars = 100 µm.

Specimens of the marine heterotardigrade *E. sigismundi* (Schultze, 1865) were sampled from barnacles at the coastline in Lynæs, Northern Zealand, Denmark (55°56′52.4″N, 11°51′08.2″E) with locality salinity and water temperature ranging between 18.1‰–23.6‰ and 1.0°C–13.9°C (Cond 3310 WTW, Germany), respectively. The tardigrades were kept on barnacle shells at approximately 6°C for up to 4 weeks in locality seawater and used in the experiments outlined below. Light microscopic images of tardigrades (Figure 1) were acquired with an Olympus DP27 digital microscope camera mounted on an Olympus BX53 microscope.

### 2.2 Experimental solutions

Six solutions constituted the basis of the experimental series: Locality seawater (Lsw) with a salinity of 18.1‰–23.6‰ depending on sampling date, ultrapurified water (∼0‰) (Barnstead EASYpure, UV/UF Control), saturated seawater (∼240‰) prepared by heating and evaporating Lsw as well as three solutions containing 1.0 mM of 2,4-Dinitrophenol (DNP) (Aldrich, Cat: D19,850-1) dissolved in Lsw, ∼0‰ and ∼240‰ solutions, respectively. DNP is a mitochondrial uncoupler that dissociates movements of electrons through the electron transport system from mitochondrial ATP production. The applied DNP concentration has previously been shown effective on marine as well as limno-terrestrial tardigrades (Halberg and Møbjerg, 2012; Halberg et al., 2013).

### 2.3 Experimental procedures

**FIGURE 2:**
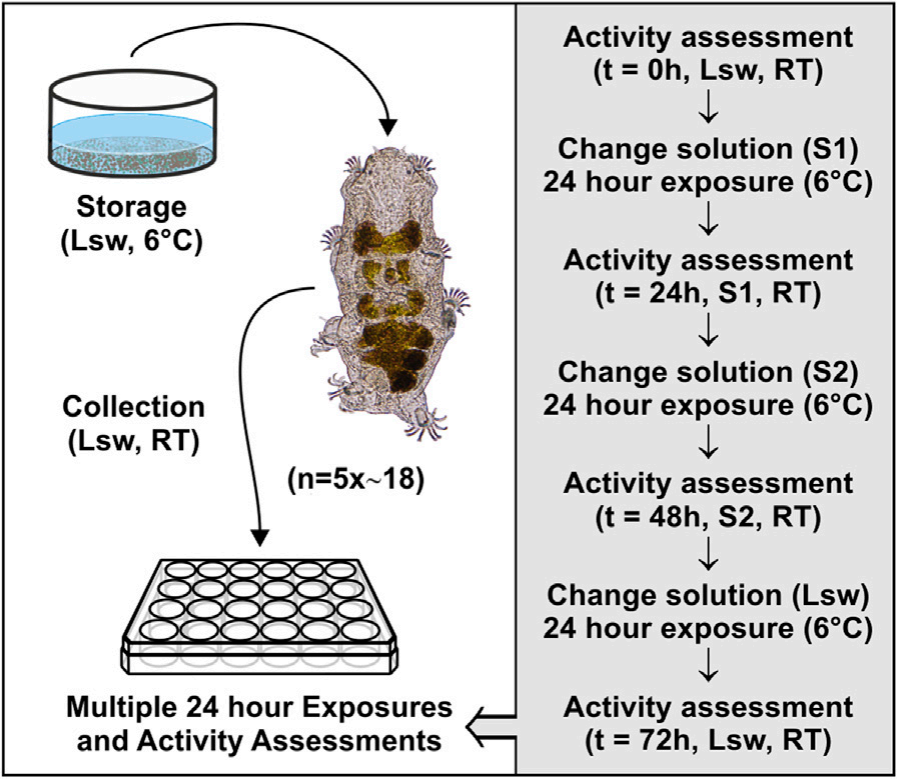
Graphical representation of the method used to evaluate the effect of exposing fully active *Echiniscoides sigismundi* to osmotic and chemical stress. Highly active tardigrades acclimated at 6°C in locality seawater (Lsw) were randomly pooled into groups (5 × ca. 18) at room temperature (RT) and transferred into the wells of a 24-well plate. The tardigrades were subsequently exposed to a given test solution (S1) and kept in this solution for 24 h before their activity was quantified. They were subsequently transferred into a new solution (S2) and kept in this solution for 24 h. Their activity was quantified and they were then transferred to Lsw for 24 h before a final activity assessment was made. Specifically, activity, calculated as the number of active tardigrades divided by the total number of tardigrades in each group, was quantified at *t = 0*, *24*, *48*, and *72 h*. S1 and S2 could be any of the six solutions constituting the basis of the experimental series (compare to Figures 3, 4). In addition to the activity assessments, the immediate response of the tardigrades towards a new solution was observed following each solution change.

Highly active tardigrades were collected from barnacle shells using a stereomicroscope (Zeiss Stemi 2000) and custom pulled Pasteur pipettes. A total of ∼740 extracted tardigrades were assigned to eight different experimental series involving transfers to the various solutions mentioned above. Sample size was defined based on previous tardigrade studies, in which significant results were obtained (*e.g.*, Hygum et al., 2016; Hygum et al., 2017; Sørensen-Hygum et al., 2018). Specifically, for each of the eight series, five groups of on average 18 tardigrades were transferred in locality seawater into the wells of a 24-well plate (TPP, Techno Plastic Products AG, Switzerland) and subsequently exposed to 1 mL of a given test solution (Figure 2). The immediate response of the tardigrades towards the new solution was observed. The tardigrades were subsequently kept in the solution for 24 h, where after their activity was quantified before transfer into a new solution. Again, the response of the tardigrades towards the new solution was observed and tardigrade activity was quantified following 24 h in the given solution. Specifically, the activity of single tardigrades was accessed at *t = 0*, *24*, and *48* *h*, with a final activity assessment at *t = 72* h, following retransfer to seawater (Figure 2). The specimens were categorized as either active or inactive. The category of active tardigrades comprised both highly active specimens and specimens with reduced activity, whereas inactive animals showed no visible movement and included swelled specimens as well as tardigrades in the tun state (Figure 1). Activity was then calculated as the number of active tardigrades divided by the total number of tardigrades in each replicate group. Following each assessment, the tardigrades were immersed into a new solution according to the given experimental series and the wells were subsequently rinsed twice with the new solution in order to remove residues of the previous test solution. The 24-well plates were transferred to a fridge at approximately 6°C in between handling (Figure 2). The activity at *t* = *72 h* was used as a measure for survival.

### 2.4 Data presentation and analyses

Data are presented as the percentage of active animals in each group at a given time point. Statistical analyses were performed by fitting a GLM (logistic regression) to the data, using a likelihood-ratio test testing for significant difference in the proportion of active tardigrades at *t = 72* *h* between selected test series. The significance level was set at *p = 0.05*. The analyses were performed in RStudio. Graphs were made in Origin Pro 9.1 (OriginLab) and final assemblage of graphs and light microscopic images was conducted in CorelDraw (Corel Corporation).

## 3 Results

### 3.1 Extreme osmotic and chemical stress tolerance in *Echiniscoides sigismundi*

**FIGURE 3:**
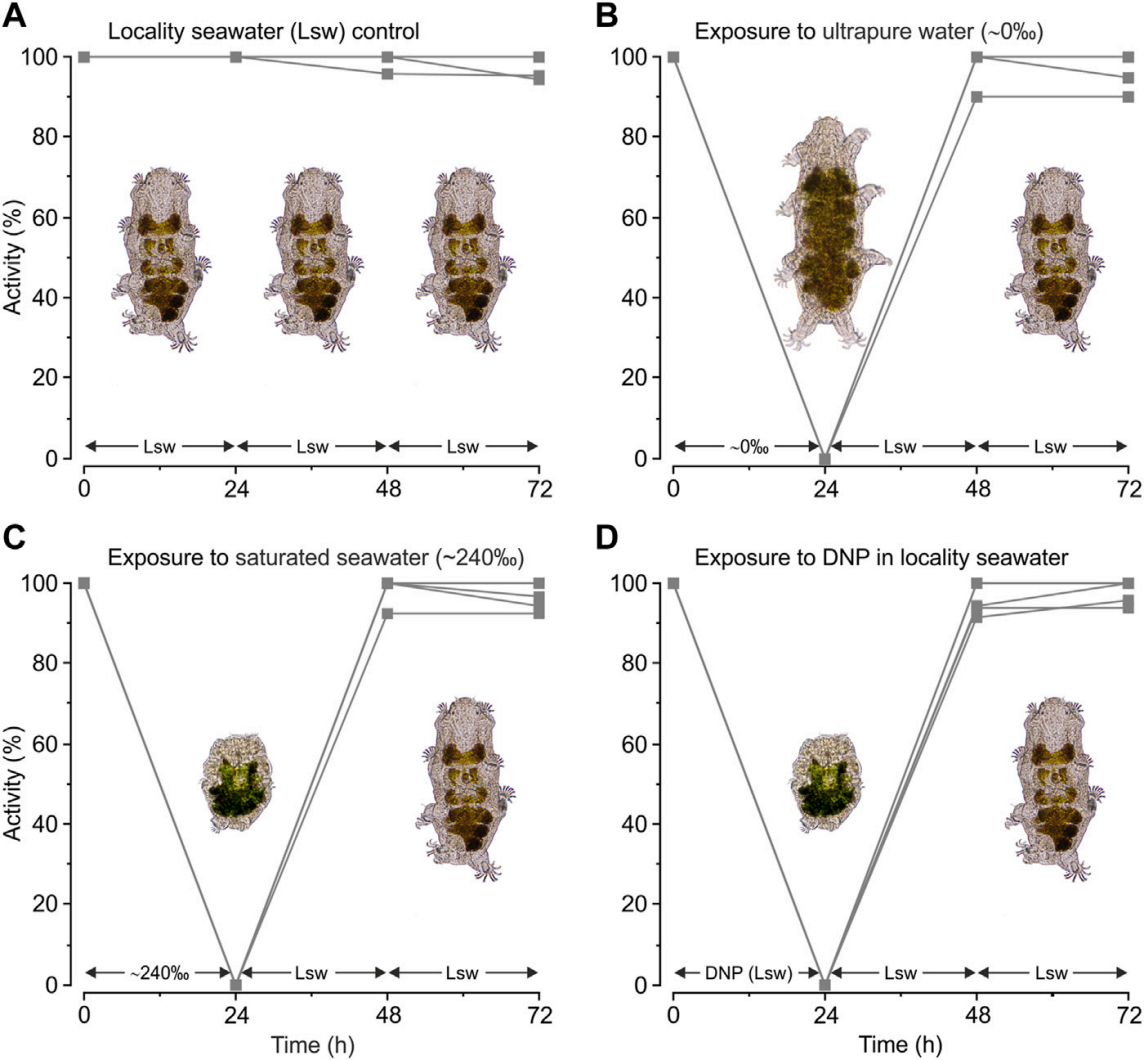
Activity of *Echiniscoides sigismundi* following exposure to osmotic and chemical stress. **(A–D)** Highly active tardigrades were kept in locality seawater (Lsw) and subsequently transferred to various solutions (compare to Figure 2). Numbers given on the *x*-axis (*0*, *24*, and *48* *h*) specify time points for activity assessment, immediately followed by change and a subsequent 24 h exposure to a new solution with final activity assessment at *t = 72* *h*. Five groups of tardigrades were used in each series. Data points reflect the percentage of active tardigrades in each group at a given time. **(A)** Activity of *E. sigismundi* under control conditions in locality seawater (Lsw). Mean ± s.e. activity at *t = 72 h* was 98% ± 1% with a median activity of 100%. **(B)** Activity of *E. sigismundi* before, during and after transfer into ultrapure water (∼0‰). Mean ± s.e. activity at *t = 72 h* was 97% ± 2% with a median of 100%. **(C)** Activity of *E. sigismundi* before, during and after transfer into saturated seawater (∼240‰). Mean ± s.e. activity at *t = 72 h* was 97% ± 2% with a median of 97%. **(D)** Activity of *E. sigismundi* before, during and after transfer into Lsw with DNP. Mean ± s.e. activity at *t = 72 h* was 98% ± 1% with a median of 100%. Activities at *t = 72 h* in the experimental series presented in Figures 3B–D are not significantly different from control conditions (Figure 3A).

Tardigrades kept under control conditions in locality seawater remained active (Figure 1A) during the entire experimental period, revealing a mean ± s.e. activity of 98% ± 1% at *t = 72* *h* (Figure 3A). When subjected to severe hypo-osmotic shock the tardigrades swelled and became immobile (Figure 1B). They remained in this swelled state for 24 h (Figure 3B, *t = 24* *h*), but they readily regained mobility following retransfer to locality seawater (Figure 3B, *t = 48* *h*) with a mean ± s.e. activity of 97% ± 2% at *t = 72* *h*. Upon exposure to saturated seawater the tardigrades also became inactive, but importantly they contracted into the quiescent tun state (Figure 1C) and stayed in this state for 24 h (Figure 3C, *t = 24* *h*). The tardigrades subsequently left the osmobiotic tun and regained full activity following retransfer to locality seawater (Figure 3C, *t = 48* *h*) displaying a mean ± s.e. activity of 97% ± 2% at *t = 72* *h*. Our results further showed that tardigrade activity dropped to 0% at *t* = *24* *h* following exposure to DNP in locality seawater (Figure 3D). Noticeably, the animals immediately contracted into the tun state following exposure to the chemical and they stayed in this state during the 24 h exposure. After return to locality seawater without DNP the tardigrades readily left the chemobiotic tun and regained activity (Figure 3D, *t = 48* *h*) with a mean ± s.e. activity of 98% ± 1% at *t = 72* *h* (Figure 3D).

### 3.2 Dissecting the mechanisms underlying osmotic and chemical stress tolerance

**FIGURE 4:**
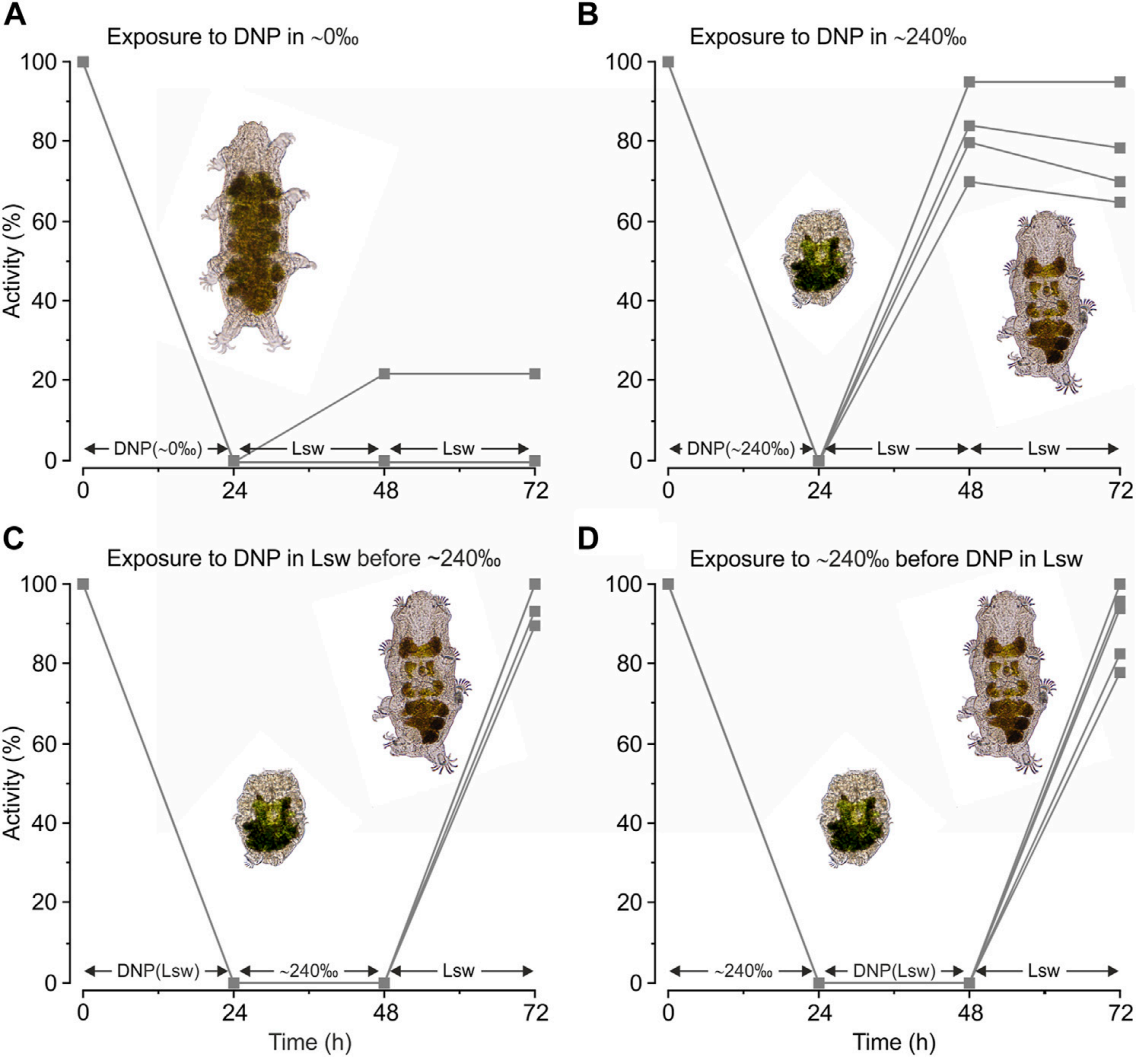
Activity of *Echiniscoides sigismundi* following combined exposure to osmotic and chemical stress. **(A–D)** Highly active tardigrades were kept in locality seawater (Lsw) prior to experimental start and subsequently transferred to various solutions (compare to Figure 2). Numbers given on the *x*-axis (*i.e.*, *0*, *24*, and *48* *h*) specify time points for activity assessment, immediately followed by change and a subsequent 24 h exposure to a new solution with final activity assessment at *t = 72* *h*. Five groups of tardigrades were used in each series. Data points reflect the percentage of active tardigrades in each group at a given time. **(A)** Activity of *E. sigismundi* before, during and after transfer into ∼0‰ with DNP. Mean ± s.e. activity at *t = 72 h* was 4% ± 4% with a median of 0%. This is a severe reduction in activity when compared to specimens exposed to ultrapure water without DNP (Figure 3B, *t = 72* *h*) (*χ^2^ = 200.95; p = 2.2 · 10^−16^*) **(B)** Activity of *E. sigismundi* before, during and after transfer into ∼240‰ with DNP. Mean ± s.e. activity at *t = 72 h* was 81% ± 6% with a median of 79%. This is significantly lower as compared to the activity following separate induction of osmobiotic (Figure 3C, *t = 72* *h*) and chemobiotic (Figure 3D, *t = 72* *h*) tuns (*χ^2^ = 12.66; p = 3.7 · 10^−4^ *and* χ^2^ = 16.61; p = 4.6 · 10^−5^*, respectively). **(C)** Activity of *E. sigismundi* following transfer into Lsw with DNP, and a subsequent transfer into ∼240‰ before return to Lsw. Mean ± s.e. activity at *t = 72 h* was 97% ± 2% with a median of 100%. This activity is not significantly different from the activity of tardigrades following exposure to saturated seawater alone (Figure 3C, *t = 72* *h*). **(D)** Activity of *E. sigismundi* following transfer into 240‰ followed by Lsw with DNP and subsequent retransfer to Lsw. Mean ± s.e. activity at *t = 72 h* was 90% ± 4% with a median of 94%. This activity is not significantly different from the activity of tardigrades following exposure to saturated seawater alone (Figure 3C, *t = 72* *h*) (*χ* ^*2*^ *=3.57; p = 0.06*).

We subsequently tested the effect of DNP on the tardigrades’ ability to handle severe hypo- and hyper-osmotic stress (Figures 4A, B). The tardigrades swelled and became immobile upon exposure to ultrapure water containing DNP and they remained in this swelled state for 24 h (Figure 4A, *t = 24* *h*). Surprisingly, most of the tardigrades did not regain activity upon return to locality seawater with a severe reduction in activity at *t = 72* *h* when compared to specimens exposed to ultrapure water without DNP (Figure 3B) (*χ* ^2^ *= 200.95*; *p = 2.2·10* ^*−16*^). This physiological response is in clear contrast to the extreme stress tolerance associated with osmobiosis and chemobiosis (Figures 3C, D). More specifically, the response indicates that tolerance towards ultrapure water (Figure 3B) may involve ATP dependent processes. The latter would imply that tolerance towards dilute solutions does not involve a reversible shutdown of metabolism and accordingly that the swelled state is distinct from the cryptobiotic state.

Tardigrades exposed to saturated seawater containing DNP became inactive and contracted into the quiescent tun state and they stayed in this state during the 24 h exposure (Figure 4B, *t = 24* *h*). The tardigrades regained activity following retransfer to locality seawater (Figure 4B, *t = 48* *h*), however, with a decreased activity at *t = 72* *h*, when compared to separate induction of osmobiotic (Figure 3C) and chemobiotic (Figure 3D) tuns (*χ* ^*2*^ *=12.66; p = 3.7·10* ^*−4*^ and *χ* ^*2*^ *=16.61; p = 4.6·10* ^*−5*^, respectively). The latter could reflect a dependency on mitochondrial ATP-production during entry into and/or exit out of the tun state. We therefore conducted two additional experimental series (Figures 4C, D) in order to further investigate a possible ATP-dependency during transition into and out of the tun state. DNP-treatment prior to immersion into saturated seawater followed by retransfer to locality seawater (Figure 4C) had no effect on activity at *t = 72* *h*, when compared to activity following exposure to saturated seawater alone (Figure 3C) (*χ* ^*2*^ *= 1.4·10* ^*−2*^ *; p = 0.91*). Tardigrades exposed to DNP immediately after saturated seawater (Figure 4D) had a mean ± s.e. survival of 90% ± 4% at *t = 72* *h*, indicating that they were challenged by ATP-depletion. The latter decrease in activity was, however, found to be above the applied significance level when compared to activity following exposure to only saturated seawater (Figure 3C) (*χ* ^*2*^ *=3.57; p= 0.06*).

## 4 Discussion

*Echiniscoides* species are common tardigrades inhabiting intertidal zones world-wide (Renaud-Mornant, 1976; Kristensen and Hallas, 1980; Hallas and Kristensen, 1982; Faurby et al., 2011; Faurby et al., 2012; Faurby and Barber, 2015; Perry and Miller, 2015; Møbjerg et al., 2016; Møbjerg et al., 2020; Gąsiorek and Kristensen, 2022). The species *E. sigismundi* has been shown to tolerate extreme fluctuations in osmotic pressure exhibiting an extraordinary resilience towards changes in external electrolyte concentration (Clausen et al., 2014; Hygum et al., 2016; Sørensen-Hygum et al., 2018). Here, we provide an experimental investigation into the phenomenon osmobiosis, using this marine intertidal tardigrade as a model. We show that the species enters the tun state and readily handles exposure to saturated seawater, and we hypothesize that osmobiosis is the forerunner of the widespread anhydrobiosis (Heidemann et al., 2016; Hygum et al., 2016; Møbjerg and Neves, 2021; Emdee et al., 2023). Notably, the echiniscoidean tardigrades, to which *Echiniscoides* belongs, have been able to cross the barrier between sea and land (Jørgensen et al., 2010; Jørgensen et al., 2011; Jørgensen et al., 2013; Gąsiorek and Kristensen, 2022).

Interestingly, DNP induced tun formation when applied to active state *E. sigismundi* in locality seawater (Figure 3D). The latter response was followed by a full regain of activity upon return to seawater without the chemical, providing evidence of chemobiosis, *i.e.*, cryptobiosis induced by environmental toxicants (Møbjerg et al., 2011). Specifically, the tardigrades entered the tun state immediately following exposure to the uncoupler and they stayed in this state during the 24 h exposure. Upon return to seawater they regained their activity. Hence, the ATP dependent muscle contraction underlying tun formation occurred before the uncoupler potentially depleted the animal of ATP. Assuming that the prolonged exposure to the uncoupler did in fact deplete ATP stores, muscle protein filaments would likely have locked in a rigor state, thereby stabilizing the chemobiotic tun, as also hypothesized for anhydrobiotic tuns (Møbjerg and Neves, 2021). Currently very few toxicological studies have been conducted within tardigrades (Sobczyk et al., 2015; Hygum et al., 2017). However, recent investigations on metal tolerance, supports the assumption that tardigrades and rotifers have the ability to enter a quiescent state in response to high toxicant concentrations (Hygum et al., 2017; McCarthy et al., 2022; Brown et al., 2023).

Marine tidal tardigrades readily cope with large fluctuations in external salt concentrations (present study; Halberg et al., 2009; Clausen et al., 2014; Jørgensen and Møbjerg, 2015; Hygum et al., 2016). In contrast, investigations by Wright and co-workers (1992) on limno-terrestrial tardigrade species revealed little survival upon transfers into 600 mOsm/kg NaCl solutions. Corresponding results on acute exposure to high NaCl concentrations were reported from other limno-terrestrial tardigrade species (Møbjerg et al., 2011; Heidemann et al., 2016). Yet, semi-terrestrial *Ramazzottius* sp. readily enter the tun state and survive transfers into high osmolality non-electrolyte solutions (∼3,000 mOsm/kg) as well as comparable osmotic pressures (∼2,000 mOsm/kg) inferred by NaCl solutions following acclimation (Heidemann et al., 2016; Emdee et al., 2023). Thus, direct transfers of non-marine tardigrades into high concentration NaCl solutions likely infer effects beyond the pure osmolytic activity of the solutions (Heidemann et al., 2016). Specifically, these tardigrades are likely sensitive towards acute perturbations in extracellular fluid [Na^+^], which may impair nerve impulses and muscle contractions, preventing tun-formation and entrance into cryptobiosis. Nevertheless, semi-terrestrial tardigrades can readily enter the tun state and survive transfers into high osmolality non-electrolyte solutions. Importantly, our present data on *E. sigismundi* indicates that survival during hypo-osmotic shock involves energy consuming processes. The tardigrades swell and become immobile when immerged into ultrapure water, and they do not tolerate the mitochondrial uncoupler DNP in this state (Figure 4A) indicating that tolerance towards very dilute solutions relies on mitochondrial energy production and that the uncoupler depleted the animals of ATP. The latter is in clear contrast to the ametabolic state of cryptobiosis. Accordingly, we stress that osmobiosis should be defined as a state of cryptobiosis induced by high osmolyte concentrations. The inducing factor is not salts (as suggested by Keilin, 1959) *per se*, but rather an increase in osmotic pressure, *i.e.*, the nature of the dissolved particles is not important—it is the associated reduction in water concentration that matters. From a thermodynamic point of view an increase in osmolyte concentration reduces the water potential of the solution and we thus hypothesize that osmobiotic and anhydrobiotic metazoans have the ability to sense this decrease and subsequently initiate the morphological and biochemical changes that prepares the organism for cryptobiosis.

A small decrease in tardigrade survival was observed following simultaneous exposure to DNP and saturated seawater (Figure 4B). This could indicate that the tardigrades are not entirely ametabolic while in the osmobiotic tun and therefore may be challenged by ATP depletion while in osmobiosis. The DNP application may also have blocked ATP-requiring steps vital for successful entrance into the osmobiotic tun state, inhibiting production of various bioprotectants, such as proteins with molecular chaperone or shielding functions or trehalose accumulation (*e.g.*, Westh and Ramløv, 1991; Schill, 2004; Jönsson and Schill, 2007; Chakrabortee et al., 2010; Hashimoto et al., 2016; Boothby et al., 2017; Hara et al., 2021; Neves et al., 2022; Emdee et al., 2023). In addition, ATP requiring antioxidant defense (Mali et al., 2010; Rizzo et al., 2010; Wełnicz et al., 2011; Jönsson, 2019; Kamilari et al., 2019) as well as ATP dependent post-cryptobiotic separation of actin-myosin cross-bridges essential for regaining muscle function (Møbjerg and Neves, 2021), may have been partly blocked by the DNP exposure. We therefore tested the effect on survival of DNP applied immediately before and after osmobiosis. Our results showed that post-cryptobiotic activity of the tardigrades were unaffected by a 24 h DNP exposure prior to the transfer into saturated seawater (Figure 4C). This result should be seen in light of DNP seemingly inducing chemobiosis, when applied in locality seawater, indicating that the tardigrades were already in a cryptobiotic state before transfer into saturated seawater. Tardigrades exposed to DNP following osmobiosis (Figure 4D) seemed to have a somewhat lower post-cryptobiotic activity, but this depression was not found to be significant in the current analyses. The nature of the small decrease in survival observed following simultaneous exposure to DNP and saturated seawater thus remains to be fully accounted for.

In summary, our data provide empirical evidence that cryptobiosis is induced by high osmotic pressure (osmobiosis) and by toxicants (chemobiosis) in the tardigrade *E. sigismundi*, whereas tolerance towards dilute solutions involves energy consuming processes. An obvious task for future studies is to provide empirical data on metabolism during osmotic and chemical induced inactivation of the tardigrades, e.g., by measuring oxygen uptake as has been done following termination of anhydrobiosis in the tardigrade *Richtersius* cf. *coronifer* (Pedersen et al., 2021).

## Data Availability

The original contributions presented in the study are included in the article/Supplementary Material, further inquiries can be directed to the corresponding author.
